# Investigation of crystal field effects for the spectral broadening of Yb^3+^-doped Lu_*x*_Y_2−*x*_O_3_ sesquioxide crystals†

**DOI:** 10.1039/d2ra03639h

**Published:** 2022-08-12

**Authors:** Ruiqi Guo, Dapeng Huang, Dazhi Lu, Fei Liang, Qingli Zhang, Haohai Yu, Huaijin Zhang

**Affiliations:** State Key Laboratory of Crystal Materials and Institute of Crystal Materials, Shandong University Jinan 250100 China dazhi.lu@sdu.edu.cn; Anhui Institute of Optics and Fine Mechanics, Chinese Academy of Sciences Hefei 230031 China zql@aiofm.ac.cn

## Abstract

The investigation of crystal field effects is significant for elucidating the spectral characteristics of Yb^3+^-doped sesquioxide crystals for ultrafast laser generation. The narrow spectra of Yb^3+^-doped single sesquioxide crystals limit the generation of ultrafast lasers; in this study, the Y^3+^ ions were introduced into Lu_2_O_3_ single crystals by the employment of ion replacement to broaden the spectra. To analyze the spectral broadening, the responsible crystal field parameters (CFPs) were calculated. The conversion of the host dominant ion and the distortion of the ligand affected the values and signs of the CFPs, and further determined the energy level splitting and fluorescence spectra. A linear relationship expressed by the semi-empirical equations for Yb^3+^-doped sesquioxide crystals was produced, which could be used for high throughput spectral prediction. Opposite variations of high- and low-frequency vibrational energies and the influence of the electron–phonon coupling on the spectra were also achieved. The redshift from the crystal field and the blueshift from the electron–phonon coupling make the optimal spectral broadening appear when *x* = 1.19 in the Yb:Lu_*x*_Y_2−*x*_O_3_ crystals. The results of these analyses could provide some key clues for the development of Yb^3+^-doped crystals for the generation and amplification of ultrafast lasers.

## Abstract 2

A linear relationship connecting the properties of composition and spectrum was deduced in Yb^3+^-doped sesquioxide crystals, and the emission spectra of Yb:Lu_*x*_Y_2−*x*_O_3_ crystals broadened under the effects of crystal field and electron–phonon coupling.

## Graphical abstract

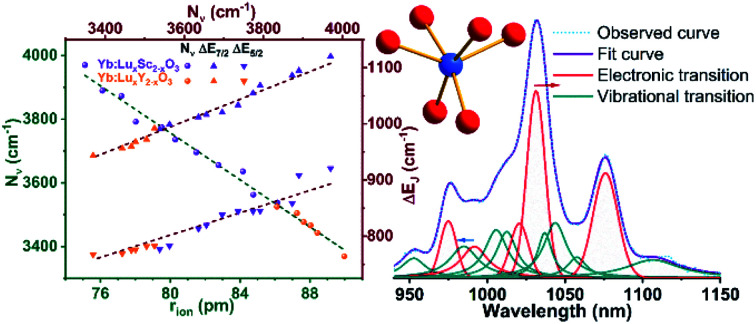

## Introduction

The crystal field theory, which describes how the crystal field influences active ions, has been used to analyze the relationship between the spectroscopic characteristics and the crystallographic structure of laser gain materials.^1–4^ The investigation of crystal field effects is demanded for Yb^3+^-based laser gain materials for a 1 μm emission, owing to the fact that energy level distributions are sensitive to crystal field interactions. The weaker shielding effect of outer 5s^2^5p^6^ for 4f^13^ shell electrons in Yb^3+^ ions enhances the effect of the crystal field compared with other lanthanide ions.^5^ A main feature of Yb^3+^-based lasers is their quasi-three-level operating scheme, in which the fundamental and terminal laser levels belong to the same ground-state manifold. In order to limit the thermal population of the terminal laser level for highly efficient laser generation, a large energy level splitting is required.^6,7^ It can be realized in Yb^3+^-doped cubic sesquioxide (Re_2_O_3_, Re = Lu, Y, and Sc) through providing a strong crystal field in comparison with various Yb^3+^-doped materials.^8,9^

The adequate spectroscopic and thermal capabilities possessed by Yb^3+^-doped cubic sesquioxides facilitate their application in ultrafast laser operations.^10–12^ The Yb:Lu_2_O_3_ thin-disk laser (TDL) generated a pulse width of 96 fs with the highest average output power of 21.1 W in the sub-100 fs regime.^13^ However, the insufficient spectral width of Yb^3+^-doped pure sesquioxide (*e.g.* Yb:Lu_2_O_3_: ∼13 nm, Yb:Sc_2_O_3_: ∼12 nm, Yb:Y_2_O_3_: ∼14 nm)^14^ limits the development of ultrashort pulse generation. The Yb^3+^-doped sesquioxide solid solutions formed by host mixing are effective for broadening the emission spectra and obtaining short pulse widths.^15–20^ C. J. Saraceno *et al.* used Yb:Lu_2_O_3_ with a full width at half maximum (FWHM) emission spectrum of 13 nm and the mixed sesquioxide Yb:LuScO_3_ with an FWHM of 22 nm to generate ultrashort pulses, and achieved shortened pulse durations from 142 fs to 96 fs.^14,21,22^ Compared with the Sc^3+^ ions, the presence of Y^3+^ ions in Yb:Lu_2_O_3_ largely retains the thermal characteristic, which is the best among these Yb^3+^-doped sesquioxide solid solutions.^23,24^ In our previous study, a ligand engineering strategy was used to disorder coordination in the Yb:Lu_*x*_Y_2−*x*_O_3_ crystal system and ultimately realized spectrum broadening.^25^ Furthermore, crystal field computations and analyses of Yb^3+^-doped sesquioxides are required for investigating the varying regularity of CFPs in the solid solutions and the mechanism of spectral broadening.

In this study, further analysis was conducted based on the results of the previous experimental research in the Yb:Lu_*x*_Y_2−*x*_O_3_ crystal system.^25^ The energy level sequence of Yb^3+^ ions in the coordinate environment was obtained from the decomposed fluorescence and absorption spectra. The CFPs were fitted with reference to the gained energy levels, and the crystal field strength and its influence on the energy level splitting and overall spectral broadening were analyzed. Meanwhile, the semi-empirical equations were obtained to predict the energy level splitting from the component of the solid solution. Furthermore, the vibrational modes obtained from Raman spectroscopy and calculated by first principles were used to analyze their influences on electron–phonon coupling and the spectral broadening mechanism. This study can serve as a basis for a deeper understanding of the crystal field effects as well as for further research on ultrafast laser gain crystal materials.

## Experimental section

The 1 at% Yb:Lu_*x*_Y_2−*x*_O_3_ (*x* = 0, 0.79, 0.99, 1.19, 1.39, 2) series crystals had been grown using the optical floating zone (OFZ) method.^25^ The fluorescence and absorption spectra of the crystals were measured at room temperature (∼25 °C) using a fluorescence spectrometer (FLS920, Edinburgh Instruments) excited at 900 nm and an ultraviolet-visible/near-infrared region spectrophotometer (UH4150, Hitachi), respectively. The observed energy levels of Yb^3+^ ions in Lu_*x*_Y_2−*x*_O_3_ crystals were obtained from the decomposed fluorescence and absorption spectra. The Raman spectra of the crystals were collected by a Raman spectrometer system (LABRAM HR-800, Horiba) excited with a 532 nm solid-state laser.

Programs for superposition model fitting (SMFit) and crystal field parameter fitting (CFPFit)^26,27^ were executed to obtain the phenomenological CFPs in the Yb^3+^-doped sesquioxide crystal system. Two fitting steps were performed: first, the SMFit program^28^ was run to obtain the intrinsic CFPs *B*_*k*_ and initial CFPs *B*^*k*^_*q*_(0) using the lattice parameters and observed energy levels. Second, the obtained *B*^*k*^_*q*_(0) were substituted into the CFPFit program as the initial values, and the final CFPs *B*^*k*^_*q*_ were fitted based on the observed energy levels using a numerical iteration method. The phonon state densities of the Lu_2_O_3_ and Y_2_O_3_ crystals were calculated using first principles based on the density functional theory,^29^ and the generalized gradient approximation with Perdew–Burke–Ernzerhof^30^ was used to describe the exchange–correlation function. The linear response method was used in the phonon calculations, and the norm-conserving method was selected as the pseudopotential.

## Results and discussion

**Fig. 1:**
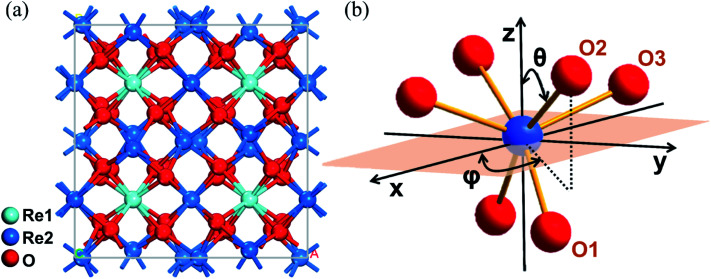
(a) Structure of the Yb:Lu_*x*_Y_2−*x*_O_3_ crystal. (b) Coordination octahedron composed of the Re2-site ion and surrounding oxygen ions in the polar coordinate system. *θ* and *φ* represent the polar and azimuth angles of each oxygen ion relative to the origin of the coordinates, respectively.

Based on the X-ray diffraction results of the grown crystals, all components of the 1 at% Yb:Lu_*x*_Y_2−*x*_O_3_ (*x* = 0, 0.79, 0.99, 1.19, 1.39, and 2) crystals exhibited the same crystallographic structure, and their point and space groups were *m*3̄ and *Ia*3̄.^25^ Their lattice parameters are list in Table S1.† As shown in Fig. 1(a), two cationic sites were found in the crystallographic structure, namely the centrosymmetric C_3i_ site denoted as Re1, and the non-centrosymmetric C_2_ site denoted as Re2. The Yb^3+^ ions occupied both cationic sites when incorporated into the crystal. Because the fluorescence was mainly derived from the electric dipole transition of Yb^3+^ ions at the non-centrosymmetric Re2-site,^31^ and the number of Re2 sites was larger than that of Re1, the cations at the Re2-site were the focus of this study. As shown in Fig. 1(b), distorted octahedron coordination was formed by the Re2-site central cation and its surrounding six oxygen ions. These oxygen ions were classified as three pairs according to the different Re–O bond lengths.

The 4f^13^ shell electrons of Yb^3+^ ion split into two manifolds under the crystal field effect when the Yb^3+^ ion is doped into the sesquioxide crystals: the ground state ^2^F_7/2_ with four Stark splitting energy levels, and the excited state ^2^F_5/2_ with three Stark splitting energy levels.^32^ The seven energy levels of each component of the Yb:Lu_*x*_Y_2−*x*_O_3_ (*x* = 0, 0.79, 0.99, 1.19, 1.39, and 2) crystals were derived according to the peak positions of the experimental fluorescence and absorption spectra. Additionally, these energy levels were used as reference values for the subsequent energy-level fitting.

In the Yb^3+^-doped sesquioxide system, the Hamiltonian is expressed as follows:^33^1
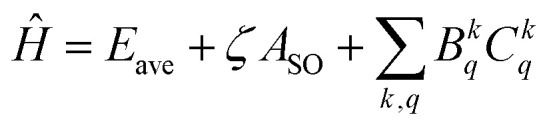
where *E*_ave_ is the average energy level, which indicates the influence of the spherically symmetric central force field; *A*_SO_ and *ζ* are respectively the angular and radial parts of the spin–orbit coupling parameter. The last term indicates the influence of the parameterized crystal field, where *B*^*k*^_*q*_ and *C*^*k*^_*q*_ represent the radial and angular parts of the CFPs, respectively. The radial parts of CFPs are impossible to calculate directly and are generally obtained by fitting. Furthermore, the residual *R* and root-mean-square deviation *σ* are calculated to measure the difference between the calculated and experimental energy levels.

**Fig. 2:**
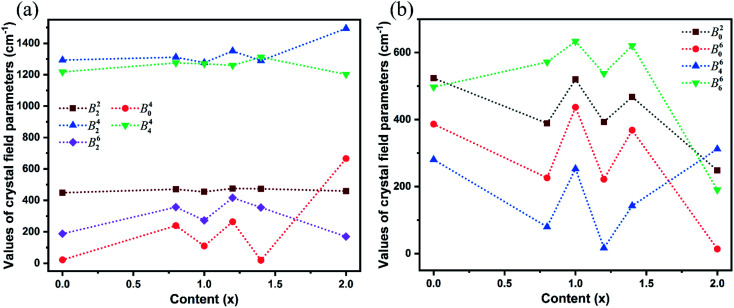
Trends of each crystal field parameter with the increased host mixing contents *x*. (a) The parameters with major contributions and the parameters of the existing local minimum at *x* = 0.99. (b) The parameters of the existing local maximum at *x* = 0.99.

The SMFit program was developed based on the superposition model,^28^ which reduced the number of initial parameters and simplified the fitting calculation. For the sites occupied by Yb^3+^ ions with C_2_ symmetry, three intrinsic CFPs *B*_*k*_ (*k* = 2, 4, and 6) were considered. Additionally, there were fifteen mutually independent parameters in the plural *B*^*k*^_*q*_, where *k* = 2, 4, and 6, and *q* was an even integer in the range of −*k* ≤ *q* ≤ *k*. The intrinsic CFPs *B*_*k*_ obtained using SMFit are listed in Table 1. The CFPs *B*^*k*^_*q*_(0), which were used as the initial parameters of CFPFit, are listed in Table S2.† For each *B*^*k*^_*q*_(0) with specific *k* and *q* values, the signs were the same for different *x* values, indicating that the influence of the coordination structure on the parameter signs was consistent. The CFPs *B*^*k*^_*q*_ obtained from the CFPFit are listed in Table 2, the corresponding experimental and calculated energy levels are listed in Table S3,† and the trends of the *B*^*k*^_*q*_ values with respect to the host mixing contents *x* are shown in Fig. 2. The fitting precision indices *R* and *σ* improved significantly after CFPFit, indicating the effectiveness of a two-step calculation. The parameter values of *B*^4^_2_ and *B*^4^_4_ were the largest, which suggested that they played a major role among the CFPs. The parameter values fluctuated with respect to *x*. As shown in Fig. 2(a), the local minimum values of each parameter were obtained at *x* = 0.99, which were the opposite of the trends shown in Fig. 2(b). The dominant ion converted at the point of *x* = 0.99 between Lu^3+^ and Y^3+^ ions in the matrix crystals, which induced the singularity of the CFPs at this component, and resulted in two opposite trends of crystal field effects.

**Table 1:** Intrinsic crystal field parameters, residuals, and *σ* obtained by SM fitting

*x*	*B*_2_ (cm^−1^)	*B*_4_ (cm^−1^)	*B*_6_ (cm^−1^)	*R* (%)	*σ* (cm^−1^)
0	992.73	875.51	333.45	0.1277	11.0843
0.79	1233.03	837.98	419.01	0.1256	10.9158
0.99	1119.73	863.38	437.74	0.1452	12.6221
1.19	1398.19	826.43	472.72	0.1174	10.2113
1.39	1166.23	869.37	448.64	0.1402	12.1989
2.00	1081.99	892.62	456.72	0.0923	8.0373

Crystal field parameters obtained by CFPFit (unit: cm^−1^)

**Table:** 

*x*	*ζ*	*B* ^2^_0_	*B* ^2^_2_	*B* ^4^_0_	*B* ^4^_2_
0	2889.37	523.47	123.4 + 431.15i	−21.37	−170.7 + 1282.18i
0.79	2889.65	388.88	−0.19 + 470.88i	−239.22	−127.29 + 1305.43i
0.99	2888.16	520.05	−26.63 + 453.72i	109.85	−118.72 + 1271.2i
1.19	2889.69	392.91	−5.56 + 475.47i	−263.97	−153.27 + 1342.5i
1.39	2888.45	467.58	−9.05 + 473.44i	18.95	−160.35 + 1279.35i
2.00	2887.11	248.09	66.61 + 454.69i	−665.65	−119.32 + 1490.08i

**Table:** 

*x*	*B* ^4^_4_	*B* ^6^_0_	*B* ^6^_2_	*B* ^6^_4_	*B* ^6^_6_
0	−1215.55 − 71.85i	386.23	−7.94 + 187.61i	278.54 + 32.82i	99.49 − 486.88i
0.79	−1268.99 − 123.78i	225.86	−335.09 + 123.66i	77.67 + 17.48i	−223.38 − 526.06i
0.99	−1264.93 − 109.76i	436.56	−159 + 221.45i	251.77 + 24.43i	40.59 − 632.21i
1.19	−1256.2 − 86.48i	221.57	−385.67 + 157.54i	−0.21 − 17.12i	−300.11 − 445.98i
1.39	−1308.91 − 88.54i	368.24	−254.76 + 247.19i	140.11 + 27.49i	−18.54 − 620.37i
2.00	−1187.26 − 188.92i	−13.95	−151.77 − 75.38i	−310.94 + 25.96i	−185.93 − 39.5i

**Fig. 3:**
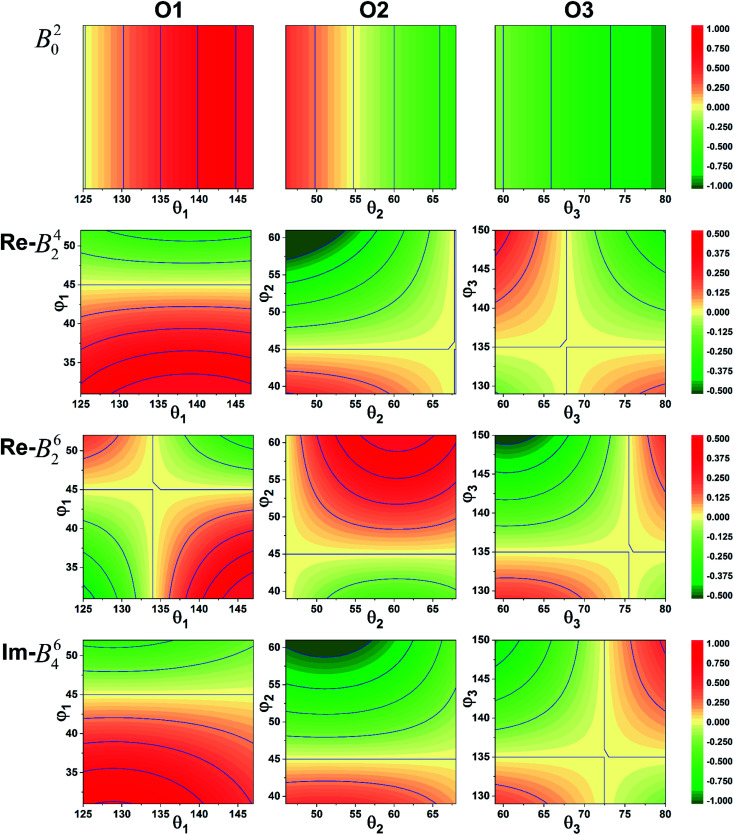
Sign variation of the CFPs with respect to the coordination ion angle *θ*_*L*_ and *φ*_*L*_ (red: positive, green: negative).

It was imperative to understand the variation in the parameter signs. Based on a comparison of the *B*^*k*^_*q*_ signs of all *x* values before and after the CFPFit calculation, as shown in Tables S2† and 2, the real and imaginary parts of *B*^4^_4_ remained negative, and the imaginary parts of *B*^2^_2_ and *B*^4^_2_ remained positive. Several signs significantly varied, such as that of *B*^2^_0_, which changed from negative to positive, whereas the signs of the real parts of *B*^4^_2_ and *B*^6^_2_ changed from positive to negative. The point charge electrostatic model (PCEM) was considered as a potential method for parsing the relationship between structures and signs. The expression for the *B*^*k*^_*q*_ parameters in the PCEM contains a summation over discrete point charges situated at the positions of the ligands:^33^2
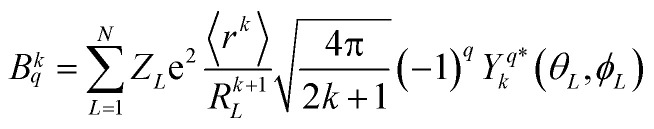
where 〈*r*^*k*^〉 is the radial integral, *Z*_*L*_e^2^ represents the multiplication of the charges of the *L*-th ligand and electron, *R*_*L*_ is the distance between the *L*-th ligand and central ion, and the subscript *L* indicates the *L*-th coordination ion. The only part that determines the sign is the angular part expanding with spherical harmonics, and the angles *θ*_*L*_ and *φ*_*L*_ of the coordination ions are the decisive factors. The spherical coordinate system was established with the central cation as the coordinate origin and the polar axis along the crystallographic *c* axis. The polar angle *θ*_*L*_ and azimuth angle *φ*_*L*_ of the coordinate oxygen ions are shown in Fig. 1(b). Assuming that each pair of O^2−^ ions has a corresponding structural distortion state, one ion from each pair of O^2−^ ions was selected as the observation object and denoted as O1, O2, and O3, respectively. According to the structural analysis results, the *θ*_*L*_ values of O1, O2, and O3 were approximately 136°, 57°, and 69°, respectively; and their *φ*_*L*_ values were approximately 42°, 50°, and 139°, respectively. Under the influence of lattice vibration, the positions of the O^2−^ ions relative to the central cation changed in accordance with the ligand distortion. Assuming that the angle deviation is within 10°, we simulated the sign part of eqn (2) and obtained the results shown in Fig. 3. As is evident from the figure, with a gradual decrease in *θ*_2_ and gradual increase in *θ*_1_, *φ*_1_, *θ*_3_, and *φ*_3_, the real parts of *B*^4^_2_ and *B*^6^_2_ tend to be negative, whereas *B*^2^_0_ and the imaginary parts of *B*^6^_4_ tend to be positive.

The crystal field strength parameter *N*_*ν*_ is a simplified description of the crystal field and is expressed as follows:^33^3
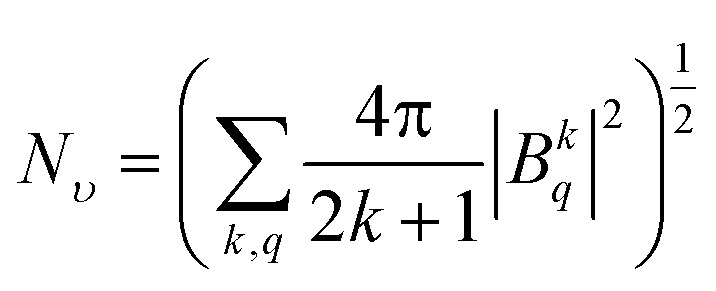


**Fig. 4:**
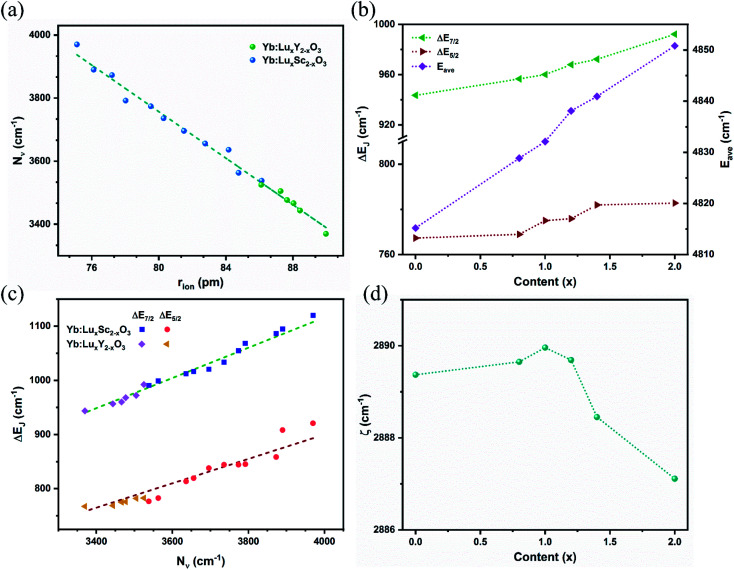
(a) Variation of the crystal field strength *N*_*ν*_ with the average ionic radius; the dashed line plots the trend of the semi-empirical formula. (b) Variation of energy levels under the influence of the crystal field with respect to host mixing contents *x*. The left *y*-axis and right *y*-axis respectively indicate the spacing of Stark splitting energy levels and the average energy levels. (c) Maxima splitting Δ*E*_*J*_ of the ground and excited states of Yb^3+^ in Lu_*x*_Sc_2−*x*_O_3_ and Lu_*x*_Y_2−*x*_O_3_ crystals varying with *N*_*ν*_. (d) Spin–orbit coupling strength varies with respect to host mixing contents *x*.

The calculated crystal field strengths of the Yb:Lu_*x*_Y_2−*x*_O_3_ crystal series are shown in Fig. 4(a). The ionic radii of Yb^3+^ [*r*(Yb^3+^) = 86.8 pm], Lu^3+^ [*r*(Lu^3+^) = 86.1 pm], and Y^3+^ [*r*(Y^3+^) = 90 pm] (ref. 34 and 35) were different, and the total cationic radius (*r*_ion_) varied with respect to the mixing content *x* of the host crystal. Given that *r*(Y^3+^) > *r*(Lu^3+^), *r*_ion_ gradually increased as *x* gradually decreased, as shown in Fig. 4(a); *N*_*ν*_ was negatively correlated with *r*_ion_. The dashed line in Fig. 4(a) is the crystal field strength line fitted by the Yb:Lu_*x*_Sc_2−*x*_O_3_ crystal series,^36^ which is expressed as follows.

4 *N*_*υ*_ (cm^−1^) = 6707.42 − 36.88*r*_ion_ (pm)

The relationship between the two crystal series was consistent, and the semi-empirical formula is suitable for various Yb^3+^-doped cubic sesquioxide crystal systems.

Stark splitting occurs at the Yb^3+^ ion energy levels under the influence of the crystal field.^5^ The differences in the split energy levels of the ground state (Δ*E*_7/2_) and the excited state (Δ*E*_5/2_) are shown in Fig. 4(b). With an increase in *x* and the crystal field strength, the extent of splitting increased. Overall, Δ*E*_7/2_ > Δ*E*_5/2_, given that ^2^F_7/2_ exhibits one more splitting energy level than ^2^F_5/2_, thus resulting in a greater difference. The average energy level *E*_ave_ of the Yb^3+^ ions with respect to *x* is shown in Fig. 4(b). Because of the energy-level expansion caused by splitting, *E*_ave_ increased with *x*. Moreover, *E*_ave_ acted as a spherically symmetric central force field in the Hamiltonian.

The crystal field strength *N*_*ν*_ is related to the energy level splitting, which has been deduced by F Auzel *et al.* using relation equations.^37^ For the ground state ^2^F_7/2_ of Yb^3+^ ions, the relation is expressed as Δ*E*(^2^F_7/2_) = 0.246*N*(^2^F_7/2_); however, the splitting of Yb^3+^ ion in sesquioxide was beyond the predictive value.^6^ Here, by combining the trends from Yb:Lu_*x*_Sc_2−*x*_O_3_ and Yb:Lu_*x*_Y_2−*x*_O_3_ crystals, two semi-empirical equations were proposed for defining the relation between the crystal field strength *N*_*ν*_ and the maxima splitting Δ*E*_*J*_ of ground (*J* = 7/2) and excited (*J* = 5/2) states of Yb^3+^ ion in sesquioxide, which are expressed as:

5 Δ*E*_7/2_ = 0.279*N*_*ν*_

6 Δ*E*_5/2_ = 0.225*N*_*ν*_

These corresponding straight lines are also represented in Fig. 4(c), and most components of the Yb^3+^ doped sesquioxide fit the trend lines well. The relations described by the semi-empirical eqn (4)–(6) can be used to predict the energy level splitting from the determined mixed content and ionic radii.

The variation in the spin–orbit coupling parameters *ζ* with respect to *x* is shown in Fig. 4(d). Theoretically, the spin–orbit coupling parameter is a free ion parameter that increases rapidly with an increase in the atomic number *Z*, and *ζ* is approximately 2900 cm^−1^ for the Yb^3+^ ion. Moreover, *ζ* varied slightly with respect to *x* and reached its maximum value at *x* = 0.99, which may be due to the influence of the central force field. Compared with the Yb^3+^-doped Lu_*x*_Sc_2−*x*_O_3_ crystal, the variation range of *ζ* was reduced for Lu_*x*_Y_2−*x*_O_3_ owing to the smaller radius difference and weaker disturbance of the central force field.

Under the crystal field effect, the splitting degree of the ground state ^2^F_7/2_ energy levels increased with increases in *x*; this caused a decrease in the spacing between the excited and terminal levels of the transition, resulting in a redshift of emission wavelength and an extension of the fluorescence spectra framework. Meanwhile, lattice vibrations and electron-coupling broadened the fluorescence spectra. Therefore, the vibrational states of the lattice were experimentally and numerically investigated.

**Fig. 5:**
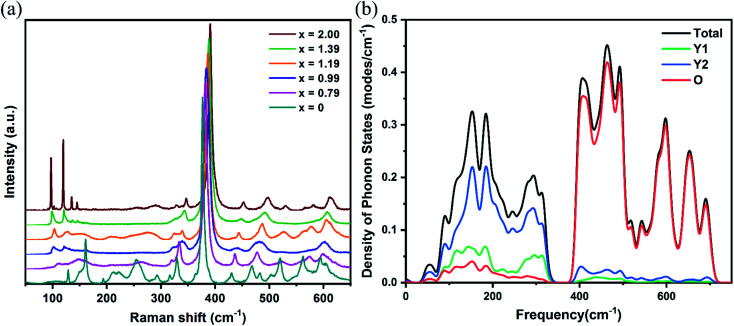
(a) Experimentally obtained Raman spectra for different *x* values. (b) Calculated density distribution of phonon states for Y_2_O_3_.

The Raman spectra of the Yb:Lu_*x*_Y_2−*x*_O_3_ (*x* = 0, 0.79, 0.99, 1.19, 1.39, and 2) crystals are shown in Fig. 5(a). The density of phonon states of the Y_2_O_3_ crystal calculated using the first-principles method is shown in Fig. 5(b). The calculated values were higher than the experimental values, perhaps because of the different conditions and the inherent defects of the computational models.^38^ As is evident from the figure, oxygen ions exhibited high-frequency (>300 cm^−1^) vibrations, which could be attributed to the stretching and bending of the Re–O bonds in the lattice. The vibrational energy was negatively correlated with the bond length that was directly related to the cationic radius. Typically, the frequency of the most intense Raman peak shifted from 376.7 cm^−1^ (Yb:Y_2_O_3_)^39^ to 391.0 cm^−1^ (Yb:Lu_2_O_3_) with *x* increasing and the cationic radius decreasing. On the other hand, the vibrations with less than 300 cm^−1^ energy were primarily from the cationic vibrations, and the cationic contribution of the Re2 site (Y2) was more significant than that of the Re1 site (Y1). It was a negative relationship between the vibrational energy and the cationic mass. As shown in Fig. 5(a), the vibration at approximately 100 cm^−1^ corresponding to the heavier Lu^3+^ ion increased with increased *x*, whereas the vibration at approximately 150 cm^−1^ corresponding to the lighter Y^3+^ ion decreased with increased *x*.

**Fig. 6:**
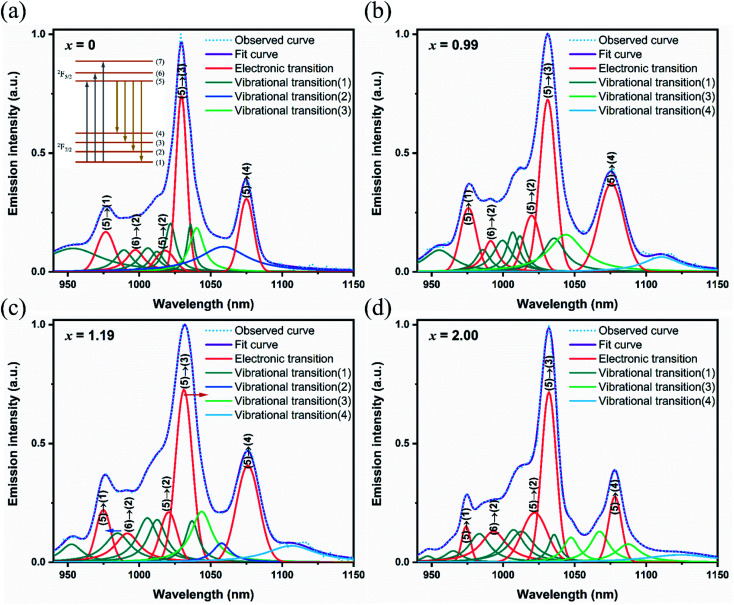
Decomposed fluorescence spectra at room temperature with different contents. (a) *x* = 0, (b) *x* = 0.99, (c) *x* = 1.19, and (d) *x* = 2.00. The electronic transition peaks have been identified as the transitions from the ^2^F_5/2_ energy levels (5)–(7) to the four ^2^F_7/2_ energy levels (1)–(4). The vibrational transitions (*i*) corresponding to the phonon sidebands of (5)–(*i*), (*i* = 1, 2, 3, and 4) energy levels transitions, respectively.

The experimental fluorescence spectra showed that the maximum broadening spectrum was 24.55 nm in the Yb:Lu_*x*_Y_2−*x*_O_3_ crystals at *x* = 1.19,^25^ which was expected to obtain pulse continuation of 45 fs.^40^ In order to distinguish the electronic and vibrational transitions, the fluorescence spectra were decomposed as shown in Fig. 6. The main transition peaks with the same positions as the spectra obtained in low temperature (77 K), researchers generally identified them as the transitions between the upper and lower energy levels which resulting from the Stark splitting.^8,41,42^ The other peaks were assigned as vibrational transitions since the enhanced electron–phonon coupling strength with the increased temperature.^43,44^ The electronic transitions were represented using Gauss line shapes because of the inhomogeneous broadening. Meanwhile, the vibrational transitions were represented using Lorentz line shapes because of the homogeneous broadening. As mentioned above, the redshift appeared on the wavelength from electronic transition with increased *x*, and the broadening from electronic transition reached the maximum at *x* = 0.99.^25^ Additionally, the lattice vibrations involved in the vibrational transition were identified. As for high-frequency vibrational transitions, the excited energy levels increased owing to the stronger crystal field, and the terminal energy levels increased owing to the higher vibrational frequency, with increased *x*. This eventually stabilized the transition peaks at approximately 1006.5, 1012.5, and 1036 nm. With respect to the low-frequency vibrational transitions, the energy level spacing was increased from the opposite changes in the excited and terminal energy levels. The blueshift was observed from approximately 990 nm to 980 nm on low-frequency transition peaks with increased *x*. The content of *x* = 1.19 for optimal spectral broadening was achieved considering the influence of the redshift from the crystal field and the blueshift from the electron–phonon coupling.

## Conclusions

In this study, the crystal field effects in Yb:Lu_*x*_Y_2−*x*_O_3_ crystals were systematically analyzed, and the intrinsic CFPs *B*_*k*_ and CFPs *B*^*k*^_*q*_ were obtained by two-step fitting. The CFPs *B*^4^_*q*_ were discovered to play a major role because of their large values among all the *B*^*k*^_*q*_. The parameter signs were affected by ligand distortion and the changes of polar and azimuth angles were discovered. Subsequently, the semi-empirical equations describing the linear relationship between the energy level splitting, crystal field strength, and the cation radius were deduced, which could be taken as a reference in Yb^3+^-doped sesquioxides research. The energy-level splitting increased along with the *x* value; consequently, the spectral framework broadened depending on crystal field effects. Based on vibrational mode experiments and calculations, the vibrations were found to participate in spectral broadening through the electron–phonon coupling process. Specifically, the high-frequency vibrations raised the intensities of the spectra and the low-frequency vibrations brought the blueshift of the transition peaks of the spectra. The findings of this study can therefore serve as a basis for the development of ultrafast laser applications based on other rare-earth ion-doped crystal materials.

## Conflicts of interest

There are no conflicts to declare.

## Supplementary Material

RA-012-D2RA03639H-s001
