# Supplementary material for: Investigation of crystal field effects for the spectral broadening of Yb3+-doped LuxY2−xO3 sesquioxide crystals
Source: RSC Adv. 2022 Aug 12;12(35):22555–63. doi: 10.1039/d2ra03639h (PMC9372876; doi:10.1039/d2ra03639h)
Supplement: RA-012-D2RA03639H-s001 [file RA-012-D2RA03639H-s001.pdf]

Electronic Supplementary Material (ESI) for RSC Advances.
This journal is © The Royal Society of Chemistry 2022

Supplementary Information

Investigation of crystal field effects for the spectral broadening of Yb^{3+}-doped $Lu_xY_{2-x}O_3$ sesquioxide crystals

Ruiqi Guo,[1] Dapeng Huang,[1] Dazhi Lu,[1] Fei Liang,[1] Qingli Zhang,[2] Haohai Yu,[1] Huaijin Zhang[1]

[1]*State Key Laboratory of Crystal Materials and Institute of Crystal Materials, Shandong University, Jinan 250100, China*

[2]*Anhui Institute of Optics and Fine Mechanics, Chinese Academy of Sciences, Hefei 230031, China*

Table S1. Lattice parameters of the Yb:$Lu_xY_{2-x}O_3$ series crystals

x	0	0.79	0.99	1.19	1.39	2.00
a (Å)	10.3776	10.462	10.4796	10.494	10.5218	10.6056

Table S2. Crystal field parameters obtained by SM fitting as initial parameters of CFPFit (unit: cm^{-1}).

x	$B_0^2(0)$	$B_2^2(0)$	$B_0^4(0)$	$B_2^4(0)$	$B_4^4(0)$
0	-98.67	129.95+316.65i	-1224.47	36.87+1351.2i	-1140.53-269i
0.79	-175.1	121.37+395.6i	-1179.04	14.33+1294.9i	-1134.49-264.08i
0.99	-151.22	115.31+353.03i	-1202.01	16.2+1335.9i	-1159.46-274.12i
1.19	-185.64	154.26+439.09i	-1153.11	19.36+1281.36i	-1112.39-263.76i
1.39	-135.08	134.63+376.5i	-1214.65	27.66+1347.57i	-1147.49-268.81i
2.00	-135.25	84.05+378.37i	-1256.62	13.7+1374.51i	-1163.36-258.5i
x	$B_0^6(0)$	$B_2^6(0)$	$B_4^6(0)$	$B_6^6(0)$	
0	133.58	11.31+32.82i	-484.89-15.05i	-68.08+59.45i	
0.79	193.03	16.12+29.76i	-622-35.73i	-73.96+82.52i	
0.99	190.77	17.08+35.09i	-641.5-34.83i	-79.91+86.6i	
1.19	206.85	17.85+39.89i	-694.97-34.98i	-90.16+93.25i	

1.39	186.29	17.64+35.43i	-654.81-26.52i	-84.66+82.52i
2.00	195.37	22.17+13.03i	-666.56-36.22i	-63.23+78.12i

Table S3. Experimental and calculated energy levels in unit cm^{-1} and calculational accuracy of CFPFit*.

x		E(1)	E(2)	E(3)	E(4)	E(5)	E(6)	E(7)	*R* (%)	*σ* (cm^{-1})
	E_{Exp}	0	422.72	532.45	943.58	10245.9	10548.52	11013.22		
0	E_{Cal}	-0.17	422.85	532.7	944.28	10246.01	10548.35	11012.38	0.0063	0.9469
	Δ_E	0.17	-0.13	-0.25	-0.7	-0.11	0.17	0.84		
	E_{Exp}	0	448.64	555.21	956.68	10256.41	10559.66	11025.36		
0.79	E_{Cal}	0	448.65	555.23	956.73	10256.41	10559.64	11025.3	0.0005	0.0683
	Δ_E	0	-0.01	-0.02	-0.05	0	0.02	0.06		
	E_{Exp}	0	452.49	558.97	960.14	10256.41	10565.24	11031.44		
0.99	E_{Cal}	-0.01	452.5	558.99	960.2	10256.42	10565.23	11031.37	0.0005	0.0787
	Δ_E	0.01	-0.01	-0.02	-0.06	-0.01	0.01	0.07		
	E_{Exp}	0	463.51	565.17	967.99	10261.67	10570.82	11037.53		
1.19	E_{Cal}	0	463.54	565.2	968.06	10261.66	10570.79	11037.45	0.0006	0.097
	Δ_E	0	-0.03	-0.03	-0.07	0.01	0.03	0.08		
	E_{Exp}	0	467.35	565.17	972.31	10261.67	10576.41	11043.62		
1.39	E_{Cal}	-0.01	467.37	565.19	972.37	10261.66	10576.38	11043.54	0.0006	0.0891
	Δ_E	0.01	-0.02	-0.02	-0.06	0.01	0.03	0.08		
	E_{Exp}	0	482.2	577.02	992.22	10266.94	10587.61	11049.72		
2.00	E_{Cal}	0.52	483.05	577.93	993.56	10266.07	10586.41	11048.2	0.0154	2.3272
	Δ_E	-0.52	-0.85	-0.91	-1.34	0.87	1.2	1.52		

$$*\Delta_E = E_{Exp} - E_{Cal}, \; R = \sqrt{\frac{\sum_{i=1}^{14}\left(E_i^{\exp} - E_i^{cal}\right)^2}{\sum_{i=1}^{14}\left(E_i^{\exp}\right)^2}}, \; \sigma = \sqrt{\frac{\sum_{i=1}^{14}\left(E_i^{\exp} - E_i^{cal}\right)^2}{3}}$$
